# Investigating the Linear Thermal Expansion of Additively Manufactured Multi-Material Joining between Invar and Steel

**DOI:** 10.3390/ma13245683

**Published:** 2020-12-12

**Authors:** Alexander Arbogast, Sougata Roy, Andrzej Nycz, Mark W. Noakes, Christopher Masuo, Sudarsanam Suresh Babu

**Affiliations:** 1 Department of Mechanical, Aerospace and Biomedical Engineering, University of Tennessee, Knoxville, TN 37916, USA; babuss@ornl.gov; 2 Manufacturing Science Division, Oak Ridge National Laboratory, Oak Ridge, TN 37830, USA; nycza@ornl.gov (A.N.); noakesmw@ornl.gov (M.W.N.); cmasuo3@gatech.edu (C.M.); 3 Materials Science and Technology Division, Oak Ridge National Laboratory, Oak Ridge, TN 37830, USA; 4 Department of Mechanical Engineering, University of North Dakota, Grand Forks, ND 58202, USA; sougata.roy@und.edu

**Keywords:** additive manufacturing, multi-material joining, Invar, steel, coefficient of thermal expansion, tooling

## Abstract

This work investigated the linear thermal expansion properties of a multi-material specimen fabricated with Invar M93 and A36 steel. A sequence of tests was performed to investigate the viability of additively manufactured Invar M93 for lowering the coefficient of thermal expansion (CTE) in multi-material part tooling. Invar beads were additively manufactured on a steel base plate using a fiber laser system, and samples were taken from the steel, Invar, and the interface between the two materials. The CTE of the samples was measured between 40 °C and 150 °C using a thermomechanical analyzer, and the elemental composition was studied with energy dispersive X-ray spectroscopy. The CTE of samples taken from the steel and the interface remained comparable to that of A36 steel; however, deviations between the thermal expansion values were prevalent due to element diffusion in and around the heat-affected zone. The CTEs measured from the Invar bead were lower than those from the other sections with the largest and smallest thermal expansion values being 10.40 μm/m-K and 2.09 μm/m-K. In each of the sections, the largest CTE was measured from samples taken from the end of the weld beads. An additional test was performed to measure the aggregate expansion of multi-material tools. Invar beads were welded on an A36 steel plate. The invar was machined, and the sample was heated in an oven from 40 °C and 160 °C. Strain gauges were placed on the surface of the part and were used to analyze how the combined thermal expansions of the invar and steel would affect the thermal expansion on the surface of a tool. There were small deviations between the expansion values measured by gauges placed in different orientations, and the elongation of the sample was greatest along the dimension containing a larger percentage of steel. On average, the expansion of the machined Invar surface was 42% less than the expansion of the steel surface. The results of this work demonstrate that additively manufactured Invar can be utilized to decrease the CTE for multi-material part tooling.

## 1. Introduction

Invar is an Fe-Ni alloy that has an exceptionally low coefficient of thermal expansion (CTE) over a broad range of useful temperatures [1]. The name Invar originates from the term invariable because of the persistent dimensional stability of the alloy over varying temperatures [2]. Between the temperatures of 30 °C and 150 °C, the CTE of Invar is between 1.2 and 2.7 μm/m-K [3]. Although Fe-Ni alloys demonstrate anomalous thermal behavior with many different compositions (some including additional components such as titanium and manganese), Invar is typically manufactured with weight percentages of 64% Fe and 36% Ni to procure the lowest possible CTE for the binary alloy [4,5]. The thermal expansion attenuation, often referred to as the Invar-effect or magnetostriction, is a result of the magnetic state of the alloy [1,6]. The ferromagnetic state required for magnetostriction only exists below the Curie temperature of Invar [7]. This temperature is a function of the composition and impurities present in the Invar alloy, and when the Curie temperature is reached, the alloy transitions from a ferromagnetic state to a paramagnetic state and loses its anomalous thermal expansion properties [8].

The low thermal expansion of Invar makes the alloy a suitable choice for many precision performance and manufacturing applications. Invar is commonly used in pipes for liquified natural gas, bimetal devices, watch hair springs, and filters for electronics [7,9]. Moreover, molds, dies, and other forming tools can be manufactured with Invar to produce equipment that maintains exceptional dimensional tolerances through forming processes that involve cyclical temperature cycles. The ability to acquire such tight tolerances is extremely beneficial to processes like composite forming in the aerospace industry. With the initiative to reduce carbon emissions and produce lighter and more efficient aircraft, the aerospace industry has begun to utilize large numbers of lightweight composite components. When molding composite parts, dissimilarities between the CTEs of resins, fibers, and the mold material can lead to geometric distortion and induce interfacial stresses and strains in the finished part [10]. The typical CTE of carbon fiber-reinforced epoxy composites is between 2.9 and 3.6 μm/m-K [11]. This is much smaller than the thermal expansion of most alloys. The CTE for typical tool steels can range from 11 to 15 μm/m-K depending on the composition of the alloy [12]. Therefore, the geometric distortion produced by an Invar mold will be far less than that of a tool steel mold. For this reason, the dimensional stability, surface quality, and durability of Invar has led to the alloy’s success and enduring popularity for composite molds [13,14].

Typically, the materials used for hard tooling include alloys of aluminum and steel [15]. These materials are inexpensive and capable of withstanding rigorous high-production cycles, but the thermal expansion of these alloys during the cyclical heating and cooling processes engenders a loss of geometric precision. Although Invar meets many of the requirements of the ideal tooling material, the alloy is relatively expensive compared to other tooling alloys (approximately 16 times the cost of carbon steel) [16,17]. Conventionally, Invar tools are manufactured through a manual process involving up to 17 or more operations; these include but are not limited to forming, butt-welding, rough machining, finish machining, and thermal stress relieving [13]. The conventional manufacturing process requires skilled personnel and involves extensive lead times. Invar is a soft and gummy alloy which makes it rather difficult to machine [4]. The inefficiency of the conventional manufacturing methods associated with the Invar alloy are further emphasized through the large amounts of material often wasted through bulk-machining processes, the excessive wear on cutting tools, and slow cutting speeds [18]. To increase the efficiency of manufacturing Invar parts, measures must be taken to optimize the amount of Invar necessary to produce the required thermal expansion properties and reduce the amount of machining required to fabricate components. 

Additive manufacturing (AM) is a process where consecutive layers of material are added on top of one another to build components directly from a computer-aided design model. Since the development of AM is the 1980s, the process has become increasingly popular because it facilitates the production of geometrically complex components and reduces lead times and human interaction [19]. Multiple metal additive manufacturing techniques have been developed to introduce the benefits of AM to the metal manufacturing industry. These techniques include powder bed fusion, binder jetting, directed energy deposition (DED), and others. Each metal AM technique can produce parts that are near net shape—significantly reducing the amount of material that must be removed by machining. Recent studies have concluded that there is between a 75% to 90% production time and cost reduction when using additive manufacturing for tooling rather than conventional methods [20]. This is particularly useful for expensive materials such as Invar. Previous research has determined that selectively laser melted (SLM) Invar maintains thermal expansion properties similar to those of conventionally manufactured Invar [4,21]. Other studies have investigated the mechanical properties of SLM Invar and suggested process parameters to achieve a stable and refined microstructure with comparable mechanical properties to the traditionally fabricated alloy [22]. Powder-bed AM processes, like SLM, can produce high-resolution parts, but the build volumes of these systems are typically less than 0.3 m^3^ [23]. Additionally, powder feedstock is very expensive, so these processes are not ideal for building large-volume components (such as molds and dies) [24]. Directed energy deposition processes have higher production rates, lower capital cost, and a larger build volume than other metal AM processes; this makes DED the most efficient and economic method for producing large near net shape components [24,25]. 

In addition to the previously mentioned benefits of DED, the industrial robots often used for DED processes can be equipped with end effectors capable of depositing two or more materials in a single layer of a build. Multi-material metal additive manufacturing has been used to successfully fabricate parts with dissimilar metals or functionally graded materials to obtain user-defined material properties throughout the part. The powder-based DED processes, such as laser metal deposition, are generally used to fabricate functionally graded materials because the powder composition can be altered between layers [26,27]. Defining deposition efficiency as the percentage of material deposited that embodies the final product, powder-based DED has a 14% deposition efficiency which is much lower than the 100% deposition efficiency attainable with wire-based DED [28,29]. Wire-based processes are not yet capable of producing functionally graded materials, but they can deposit dissimilar metals to achieve local material properties throughout the part [30]. Dissimilar metal joining using high energy-density beams has been used to improve joint characteristics and reduce distortion in additively manufactured parts. Hinijos et al. utilized electron beam melting additive manufacturing to join Inconel 718 and 316 stainless steel [31]. They noted that the affinity of the dissimilar alloys to precipitate and form phases or precipitates can have undesirable effects on the weld quality or material properties [31]. Additionally, they observed cracks at the interface of the Inconel and stainless steel due to the dissimilarity between the CTE of the substrate and the filler material [31]. T. Abe and H. Sasahara used wire-arc based additive manufacturing to join stainless steel and a nickel-based alloy to produce a part with high heat and corrosion resistance [28]. Using energy dispersive X-ray spectroscopy, the authors found that the nickel alloy had been diluted with iron from the stainless steel at the interface of the two materials [28]. They claimed that the dilution would reduce the resulting materials heat and corrosion resistance, but the mechanical properties of the combined alloys proved comparable to those of the individual materials [28]. For Invar, the multi-material additive manufacturing process is a promising technique for reducing the amount of Invar needed to obtain the required thermal properties. Parts can be manufactured with an Invar shell and an inner metal core (such as low-carbon steel) to tailor the thermal expansion properties to specific applications while enhancing the economic feasibility of Invar fabrication. 

Multiple studies have analyzed methods for joining Invar and dissimilar alloys. Xu et al. used tungsten inert gas (TIG) welding to join Invar and carbon steel plates [32]. They noted that residual stresses caused by thermal expansion coefficient mismatches and second-phase precipitates from element diffusion are the two primary concerns associated with dissimilar metal joining involving Invar. They investigated the effects of temperature field distribution and its relation to element diffusion and concluded that a full penetration dissimilar weld joint between Invar and steel was obtained using their specified welding parameters. Li et al. successfully joined two Invar 36 plates using ER309 filler wire [33]. They used both laser welding and laser-arc hybrid welding to compare the microstructure, thermal expansion characteristics, and mechanical properties of the weld joints. The authors claim that the base material and the laser welded specimens match closely to the theoretical expansion expected from the Invar alloy, but the laser-arc hybrid welded specimen exhibits a sharp increase in CTE. They extended this explanation and described that hybrid welding resulted in an alloy with a smaller fraction of the γ-(Fe,Ni) phase—the phase responsible for the Invar effect. The most popular 2γ-state model for the Invar effect proposed by Weiss states that the thermal expansion attenuation is a function of both the ferromagnetic high-volume state and antiferromagnetic low-volume state of the alloy [1]. Li et al. reported that the hybrid welding process resulted in a decreased Ni percentage and the introduction of elements such as C, Mn, and Cr. The reduction in nickel percentage and the addition of the alloying elements produced a new phase of FeCr0.29Ni0.16C0.06 resulting in a two-phase alloy that has a smaller fraction of γ-(Fe,Ni) and did not exhibit the same thermal expansion characteristics as the single-phase γ-(Fe,Ni) alloy.

In the present study, Invar sections of varying thicknesses were deposited atop a steel base plate and samples were taken from below the Invar beads, from the weld interface, and directly from the printed bead. The samples were used to investigate the thermal expansion properties throughout the welded specimen. Energy-dispersive X-ray spectroscopy (EDS) and electron micro probe analysis (EPMA) were performed to analyze the composition of the alloy along the interface regions of Invar and the base plate. The study found that steel samples taken from under the Invar bead had CTEs that were typical for steel. The diffusion in and around the fusion zone of the weld increased the CTEs for most samples taken from the interface of the two materials. The samples taken directly from the Invar bead showed a decreased thermal expansion, and the diffusion had a smaller effect when the samples were taken farther from the fusion zone. In addition to finding the local thermal expansion properties of the printed specimen, the nominal surface expansion of a part was analyzed to determine how well the part would function in low thermal expansion applications. Invar beads were deposited on a steel base plate, and the beads were face milled to replicate the surface of a post-processed additively manufactured tool. The part was then heated in an oven, and strain gauges were used to measure the overall thermal expansion of the combined materials. The surface expansion of the face-milled Invar beads was measured to be between the expected thermal expansions for Invar and steel.

## 2. Materials and Methods 

**Figure 1:**
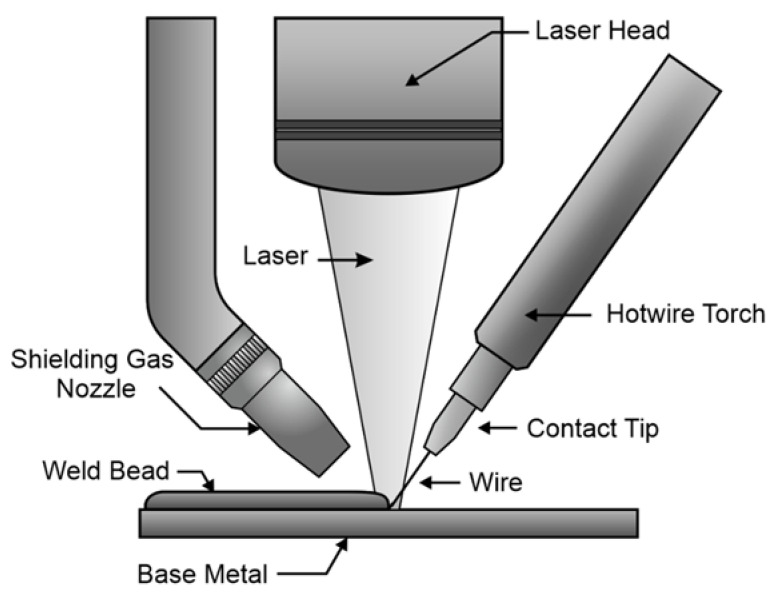
Laser end-effector used for the laser-hot wire additive manufacturing process.

Invar beads were deposited on two 9.5 mm-thick A36 steel base plates using a laser-hotwire additive manufacturing process. The laser-hotwire process uses a laser end-effector held by an industrial robot. The laser end-effector is moved while wire is fed through a hot wire torch, and the laser melts the filler wire to deposit a weld bead. A schematic of the laser hot wire process is provided in Figure 1.

Aperam Invar^®^ M93 wire was used to deposit invar beads on both the first and second steel plates. The chemical composition of the wire provided by the vendor is shown in Table 1.

**Figure 2:**
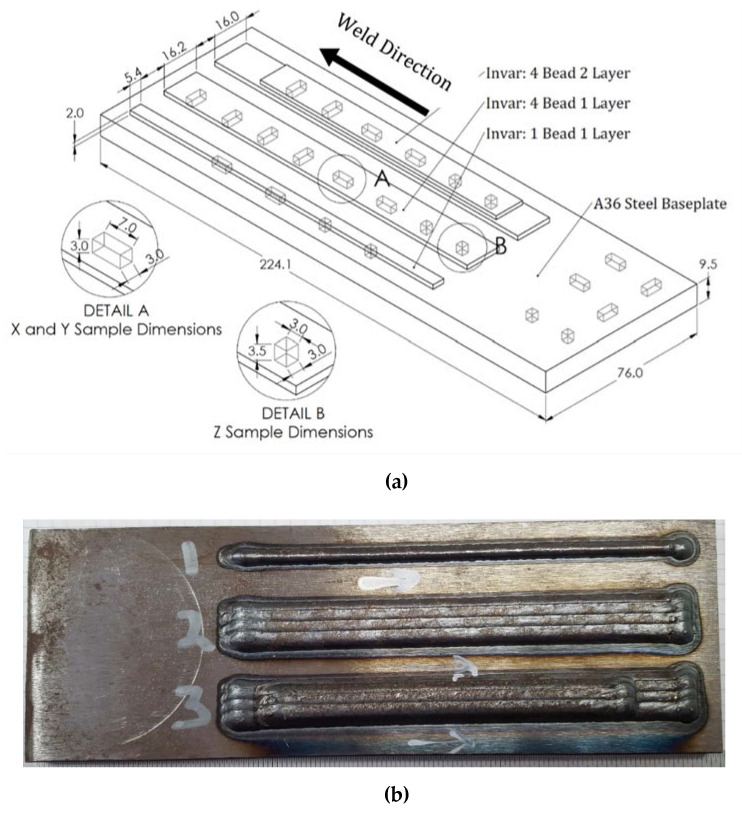
(**a**) Invar bead layout and Invar sample locations on A36 steel baseplate and measured dimensions (mm) of base plate and samples in each direction. (**b**) Welded invar beads on steel baseplate before sample removal.

The first welded plate contained three sections varying in both the number of beads and layers of Invar. The approximate dimensions of the invar beads and the base plate are provided in Figure 2a, and the physical specimen is shown in Figure 2b. The invar deposited on the second plate was two beads in width, and the weave amplitude was increased to 10 mm make the width of each bead approximately 20 mm. The beads were spaced 19 mm apart two ensure a 1 mm overlap between beads. Two passes for each bead were performed, and the final height of the beads was approximately 4 mm. The plate was cut to 230 mm in length and 30 mm in width, and the top of both beads on the second plate were machined to leave a smooth and flat 2.8 mm-thick surface for thermal expansion analysis. The Invar beads on both plates were deposited using 1.2 mm Invar M93 wire and a wire-based Lincoln Electric fiber laser system. Argon was used as the shielding gas for the welding process, and the laser power was set to 5800 W. Robot travel speeds for welding the first and second plates were 21.2 mm/s and 5.1 mm/s, respectively, and weaving was used on the second plate to produce a wider bead.

**Figure 3:**
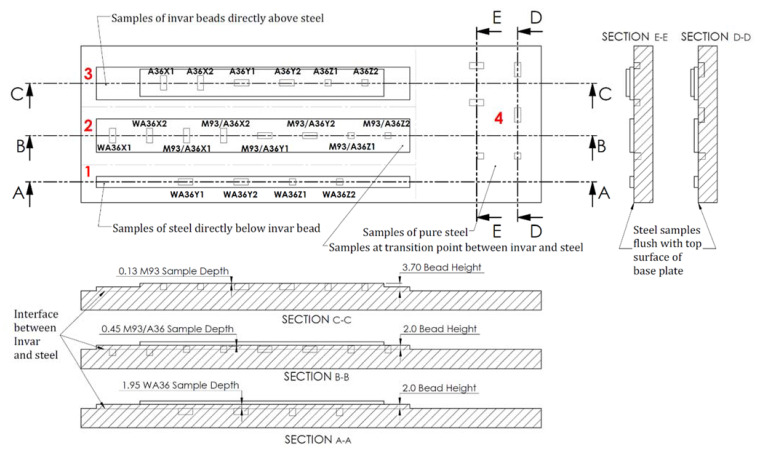
A36 steel baseplate and welded Invar bead sample locations for thermomechanical analysis and corresponding cross-sectional views of each Invar and steel section with the respective sample depths in relation to the material interface and top of the weld beads.

Samples were machined from four sections (indicated 1, 2, 3, and 4 in Figure 3) on the first welded plate. The dimensions of samples taken from the X, Y, and Z directions can be found in Figure 2a, and the sample distance from the top of the weld bead as well as the distance from the interface of the two materials can be inferred from Figure 3. Section 3 has two layers of Invar, and sections 1 and 2 have one layer of Invar. Samples are referred to according to their material, direction, and number. The label M93 refers to samples taken from a two-layer Invar bead. M93/A36 refers to samples taken from the interface between the Invar bead and the steel substrate, and WA36 refers to samples taken from the substrate under the Invar bead. Samples taken from the pure steel region of the baseplate are labeled as A36. M93, M93/A36, and WA36 samples were taken from 0.13 mm, 0.45 mm, and 1.95 mm below the surface of the Invar bead, respectively. A36 steel samples were taken from the top surface of the steel.

Selected samples from the first plate were studied using energy-dispersive X-ray spectroscopy (EDS) to analyze elemental distribution along the weld metal and base metal interface regions. For that, metallographic specimens were prepared by sectioning the weld interface regions using a precision saw and mounted on conductive bakelite. Later, they were prepared using standard metallographic procedures of up to 1 µm diamond paste polish. Scanning electron microscopy (SEM) and EDS spectroscopy of the interface regions were conducted using a JEOL 6500 FEG-SEM (JEOL USA, Inc., Peabody, MA, USA) at 20 kV equipped with EDAX and electron back scatter diffraction (EBSD).

Compositional analyses using EPMA were acquired on an electron microprobe (JEOL-JXA8200) (JEOL USA, Inc., Peabody, MA, USA) equipped with five tunable wavelength dispersive spectrometers. EDS spectra were acquired and processed using a Thermo Pathfinder EDS (Thermo Fisher Scientific., Waltham, MA, USA) system. The operating conditions were a beam energy of 15 keV and beam current of 50nA. Elements were acquired using EDS for Fe Kα, Ni Kα, and analyzing crystals LIFH for Mn kα, and TAP for Si kα. The counting time (both on peak and off peak) was 10 s for both Mn and Si. The off-peak correction method was linear for Mn and exponential for Si. Standard intensities were corrected for standard drift over time. The width of the line scans was 1µm to avoid capturing signals from sides which could affect the data.

A Q400 thermomechanical analyzer (TMA) (TA Instruments, New Castle, DE, USA) was used to measure the thermal expansion for samples taken from the first plate. The initial height of each sample was measured using the TMA’s thermal expansion probe, and the probe was set to expand from the initial height of each sample. Samples were held at 40 °C for five min to maintain an isothermal temperature profile. The temperature was increased from 40 °C to 150 °C at a rate of 5 °C/min, and thermal expansion measurements were taken at a frequency of 2 Hz. The coefficient of thermal expansion α for each sample is given by:

(1) $$\alpha =\frac{\Delta L}{L_{0}\Delta T}.$$

Using Equation (1), the CTE of each sample was calculated with the original length L_0_, change in length ∆L, and change in temperature ∆T recorded from the TMA.

**Figure 4:**
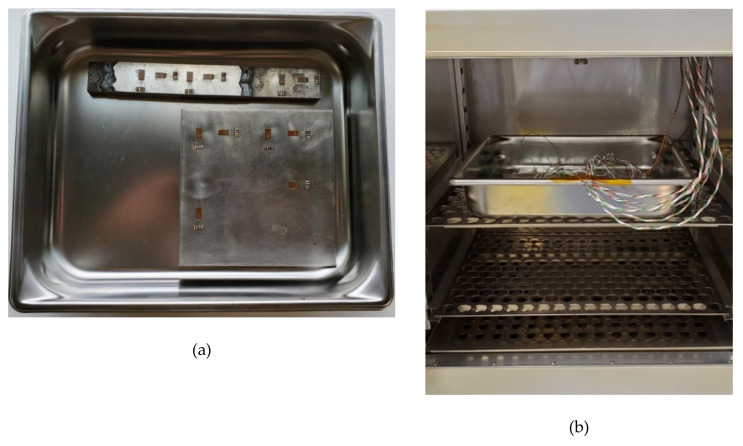
(**a**) Strain gauges bonded to the machined Invar beads, steel, and Invar reference plate. (**b**) Invar plates in the oven with strain gauges and thermocouples.

The second welded plate and an invar reference plate were used to analyze surface expansion of the machined Invar beads. The second welded plate, the invar reference plate, and the experimental setup is shown in Figure 4. The surface expansion of the additively manufactured and machined specimen was measured to determine how the combination of materials would affect the overall thermal expansion on the surface of a part. The thermal expansion on the surface of the steel and the invar was measured using 12 strain gauges (forming six half-bridge circuits).

**Figure 5:**
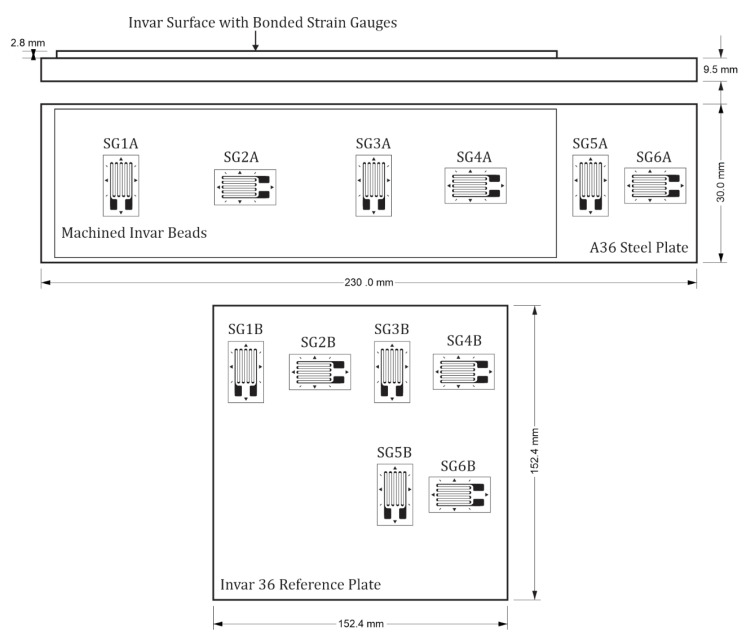
Location and orientation of strain gauges on the machined invar beads, steel plate, and the invar reference plate. Dimensions of each plate and the interface between the steel and Invar.

Strain gauges are sensors that have a change in resistance proportional to the strain induced on the surface of the gauge. In the case of thermal expansion, this strain is also commonly referred to as the thermal output [34]. The 12 gauges were bonded in pairs with one gauge on the analyzed specimen and the other on an Invar reference plate. When the surface expansion is measured with a half-bridge strain gauge configuration, the thermal expansion will be the difference between the individual thermal outputs of the strain gauges on the machined Invar beads and the Invar reference plate [34]. WK-00-125AD-350 strain gauges (Micro-Measurements—Vishay Precision Group, Malvern, PA, USA) were used to compare the thermal expansion of the machined Invar beads and the steel to the expansion of the Invar reference plate. The location and orientation of each strain gauge is shown in Figure 5. The printed invar extends to the edges of the baseplate in the physical specimen; however, space is left in Figure 5 to distinguish the baseplate from the printed beads.

The WK-00-125AD-350 strain gauges were fully encapsulated K-alloy gauges that were chosen because of their minimal self-temperature compensation that matches the low thermal expansion coefficient of Invar [35,36]. The reference plate measured 152.4 mm × 152.4 mm × 2.5 mm, and the CTE of the plate met the American Society for Testing and Materials (ASTM) F1684 specifications [3]. M-Bond 610, a strain gauge adhesive with a maximum operating temperature of 260 °C, was used to apply the strain gauges to the steel, Invar, and the reference plate to ensure that the oven temperature did not exceed the maximum operating temperature of the adhesive [37].

**Figure 6:**
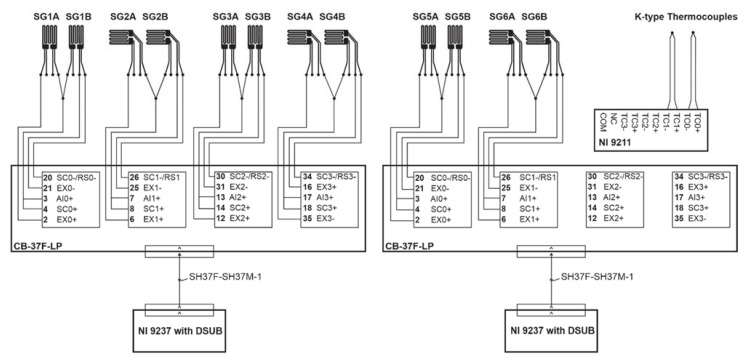
NI 9237 strain gauge module and NI 9211 thermocouple module wiring diagram.

K-type thermocouples were used to measure the temperature of each plate. A 1/16 in. hole was drilled in the base of the plates, and thermocouples were inserted into the holes. A National Instruments 9211 temperature input module was used to record temperature data from the thermocouples, and the strain gauge measurements were recorded using a National Instruments 9237 bridge completion module. Each module was inserted into a National Instruments CompactRIO backplane, and a LabVIEW program was used to collect the data. The wiring diagram for each module is shown in Figure 6.

The assembly was placed inside a DX402C oven (Yamato Scientific Co., Ltd., Santa Clara, CA, USA) (shown in Figure 4b), and ramped from 40 °C to 160 °C. The temperature of the oven was ramped at 20 °C increments, and strain gauge data were taken at each temperature increment when the thermocouples indicated a homogeneous temperature between the plates. After collecting the data, the CTE of the Invar surface was determined by:

(2) $$\alpha_{surface}=\frac{\left(\varepsilon_{surface}-\varepsilon_{ref}\right)}{\Delta T}+\alpha_{ref},$$

where $\varepsilon_{surface}$ is the strain on the machined Invar bead surface,$\;\varepsilon_{ref}$ is the strain on the Invar reference plate, and $\alpha_{ref}$ is the manufacturer’s specification for the CTE of the Invar reference plate [34]. The measurement acquired from the NI 9237 is the amplitude ratio of the output voltage (measured over the Wheatstone bridge) and the excitation voltage. The voltage ratio is converted to a strain differential with the following:

(3) $$\varepsilon_{surface\;}-\varepsilon_{ref}=\frac{2V_{out}}{V_{ex}}.$$

As an additional test, the experiment was repeated to measure the CTE of the Invar reference plate and a low-carbon steel plate. Two half-bridge strain gauge circuits were used to measure these CTEs. The first half-bridge had one gauge mounted on the low-carbon steel plate and the other mounted on the Invar reference plate. The other half-bridge had both gauges mounted on the Invar reference plate. This additional test was performed to confirm that the CTE of the reference plate was within the expected values of 1.2 and 2.7 μm/m-K, and the A36 steel plate with a known CTE was used as an experimental control [38].

## 3. Results and Discussion

### 3.1. Thermomechanical Analysis and Energy Dispersive X-ray Spectroscopy on Different Samples 

The CTEs for samples in each section of the specimen (refer to Figure 3) are provided in Table 2. The average CTE of each section is included to demonstrate how the various thicknesses of Invar and the sample locations affected the overall thermal expansion of the section.

**Figure 7:**
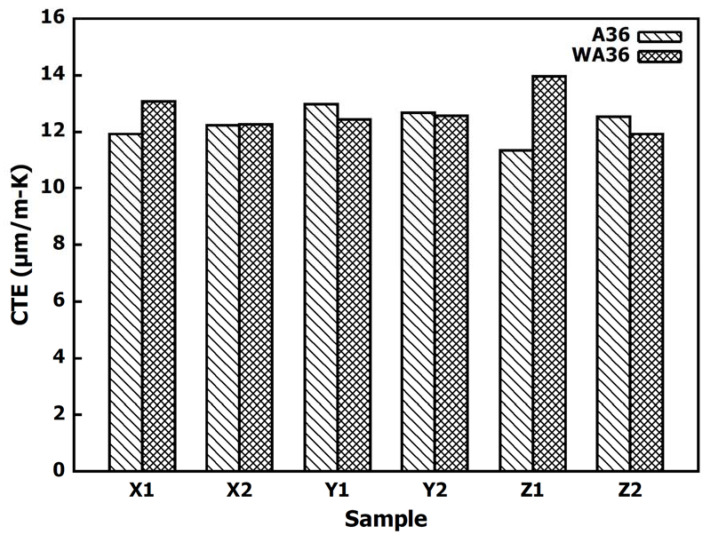
CTE comparison between the samples taken from the top surface of the steel and samples taken from 1.95 mm under the top of the invar bead.

The A36 samples taken from the steel region of the baseplate had thermal expansion coefficients close to the value of 12.0 μm/m-K expected for A36 steel [12]. The measured expansion data indicate that the steel samples had isotropic thermal characteristics with an average CTE of 12.27 ± 0.58μm/m-K, (standard deviation is denoted by ±). The A36 CTE values are used as a reference to compare the CTEs measured in the each of the Invar sections. The WA36 steel samples, taken from under the one-layer Invar bead, behaved similarly to the A36 samples and had an average CTE of 12.69 ± 0.73 μm/m-K. The CTEs for the WA36 samples are plotted against the CTE of the A36 samples in Figure 7.

The WA36 samples have a larger variation between the CTEs measured in each direction than those of the A36 samples—as indicated by the larger standard deviation. When the WA36 samples were removed from the baseplate, the cutting depth was measured with respect to the top of the plate. Hence, the top face of each sample is flush with the top face of the base plate. Although the weld penetration depth should remain relatively consistent, the penetration depth was assumed to have minor fluctuations along the length of each bead. Depending on where the sample was taken from the bead, certain samples may have contained a greater percentage of Invar than others. Also, since steel is considered to have isotropic thermal expansion properties, the disparity between CTE values can be considered a function of the sample location along the bead rather than the sample direction. In many of the previous studies conducted on dissimilar metal joining, the authors stressed the importance of element dissolution in the heat affected zone (HAZ) during the welding process—noting that it had measurable effects on the thermal and mechanical properties of the resulting alloys. While the WA36Y and WA36Z samples were taken from under the single Invar bead (section 1 in Figure 3), the WA36X samples were taken from under the three beads in section 2. This was necessary because the single bead was not wide enough to take samples in the X direction. However, printing three beads, instead of one, leads to a larger HAZ as well as a greater chance for element diffusion in section 2 of the specimen. The WA36X1 sample was also taken from the start of the bead where the initial laser power must be higher to begin melting the material. This often leads to a greater weld penetration depth and a higher temperature near the ends of the beads. These factors lead to a greater chance of dissolution at the interface of the materials and lend a potential explanation for the greater CTE seen in the WA36X1 sample. The mechanisms by which diffusion contributes to an increased thermal expansion is discussed later.

**Figure 8:**
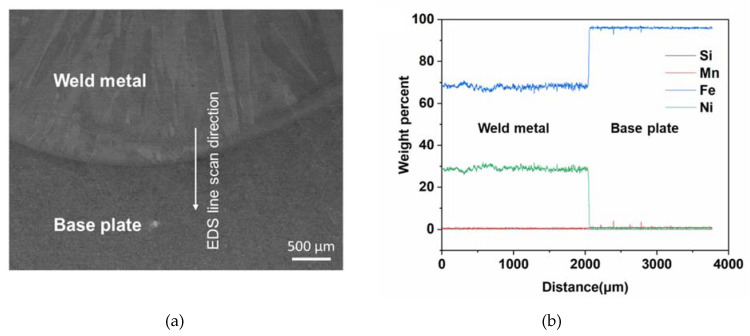
(**a**) Secondary scanning electron image of interface region and (**b**) energy-dispersive X-ray spectroscopy (EDS) line scan signals along the interface for the WA36X1 sample.

Figure 8 shows the secondary scanning electron image of the weld metal-base plate interface region and the EDS line scan signals along the interface. The key constituting elements were Si, Mn, Fe, and Ni, but the weight percent of Si and Mn was negligible (below 1%). Ni percentage also dropped close to zero after the interface and Si, Mn and Ni signals overlapped each other. The Ni percentage below 36% (the optimal percentage for the lowest CTE in Invar) resulted in a significant increase in CTE of the WA36X1 sample.

**Figure 9:**
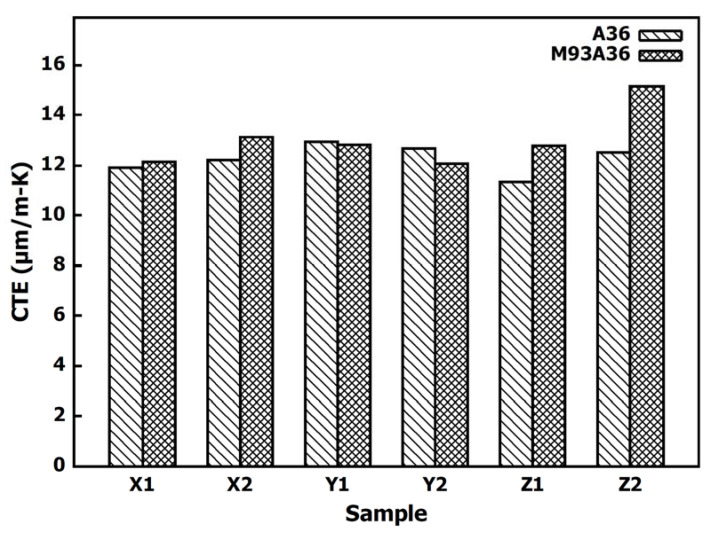
CTE comparison between the samples taken from the top surface of the steel and samples taken from the interface between the steel and the invar bead (0.45 mm below the top of the Invar bead).

The M93/A36 samples (taken from the interface of the Invar and steel) demonstrated a linear thermal expansion similar to that seen in the WA36 samples. No signs of an Invar-like expansion curve are seen in the first two sections of the samples. The average CTE of the M93/A36 samples is 13.02 ± 1.12 μm/m-K. A comparison of M93/A36 and A36 sample CTEs is shown in Figure 9.

Four out of six of the Invar sample CTEs in the M93/A36 section are larger than the sample CTEs from the steel region. The effects of dissolution are most noticeable in the M93/A36 section where samples were taken from the fusion zone of the weld. Like the WA36X1 sample that was taken from the end of the three beads, the M93A36Z2 sample on the opposite side of the beads shows a significant increase in thermal expansion. The samples taken closer to the center of the bead exhibit a smaller thermal expansion while those taken from the end regions show a sharp increase in the CTE. The samples in the Z direction were also half of the volume of the samples taken from the X and Y direction to ensure an equal area of material was taken from the fusion zone. Therefore, any increase in CTE that is caused by the diffusion process is likely to have a greater effect on the expansion of the samples with a smaller volume. The average expansions of the WA36 and M93A36 sections indicate that the element diffusion had the greatest impact on increasing the CTE closer to the fusion zone of the weld. 

**Figure 10:**
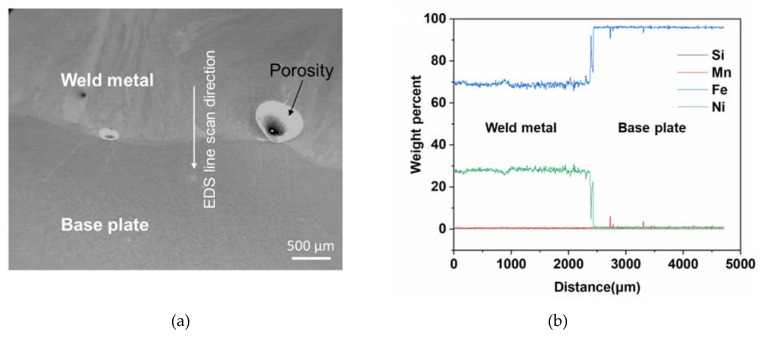
(**a**) Secondary scanning electron image of interface region and (**b**) EDS line scan signals along the interface for the M93A36Z2 sample.

Figure 10 presents the secondary electron micrograph of the weld interface region along with the EDS line scan captured from the M93A36Z2 sample. It can be observed that the Ni percentage for this sample is even lower (average weight percentage 28%) with a slightly higher Fe percentage compared to that of the WA36X1 sample due to dissolution of iron during the fabrication process. The low Ni percentage resulted in a significant increase in the CTE for the M93A36Z2 sample.

**Figure 11:**
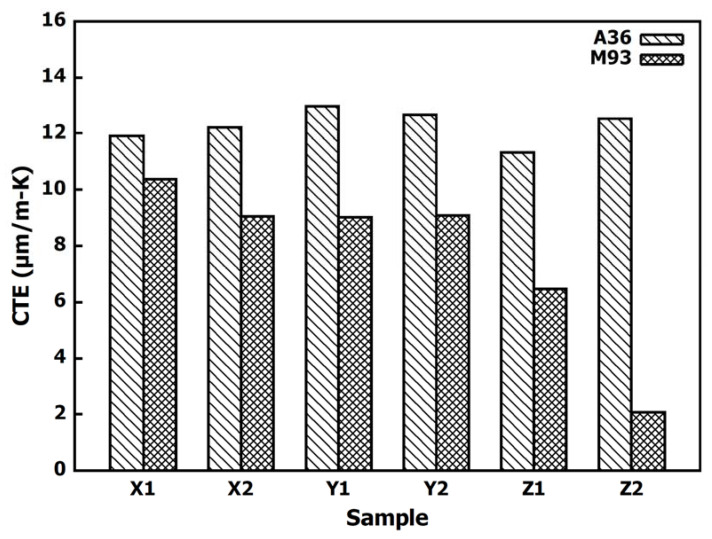
CTE comparison between the samples taken from the top surface of the steel and samples taken from 0.13 mm below the top of the Invar bead.

The CTEs measured from the two-layer Invar section are much lower than those measured in the previous sections. The average M93 sample CTE is 7.69 ± 3.02μm/m-K. A comparison of the CTEs for samples taken from the M93 and A36 sections is provided in Figure 11.

The M93 section was the only section where all the sample CTEs remained below the CTE of the A36 steel samples. Since these samples were taken from above the weld interface, they were less likely to experience the effects of diffusion observed in the other sections of the specimen. The CTEs of samples generally decreased from one end of the bead to the other. As observed from the previous section, the M93X1 sample with the highest CTE was taken from the end of the bead. Conversely, the M93Z2 sample had the lowest CTE and was also taken from the end of the bead. The sample with the lowest CTE was never located on the end of a bead in any of the previous sections. Since this section had two layers with three beads per layer, the broader distribution of sample CTEs is likely an effect of the heat exposure patterns during the welding process. Each sample contained enough Invar to maintain a CTE lower than that of steel, but the section of the beads closer to the X-direction samples was likely exposed to more heat than the section of the bead near the Z-direction samples. The diffusion that resulted from the increased heat exposure altered the thermal expansion properties and increased the CTE of the samples in a similar manner to that seen in the M93A36 section. However, the volume of the samples that was affected by the diffusion was not large enough to increase the CTE past that of steel. 

**Figure 12:**
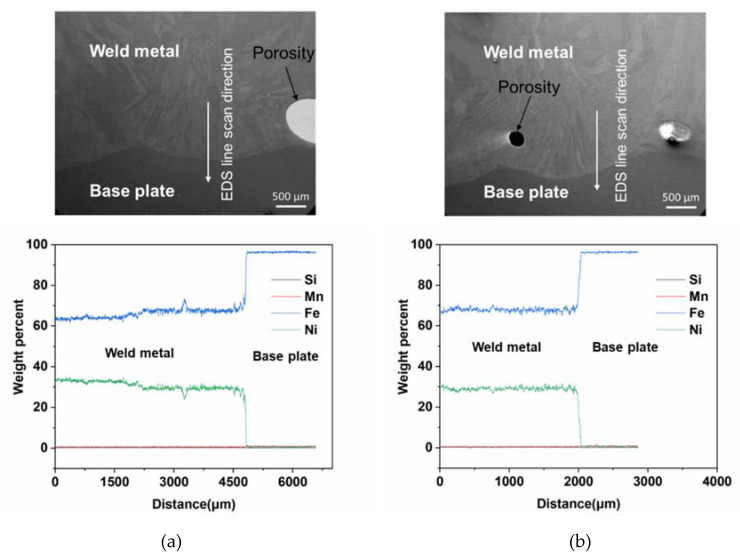
Secondary scanning electron image of interface region and EDS line scan signals of (**a**) M93Z2 and (**b**) M93X1 samples.

Figure 12a,b shows secondary image and EDS line scan signals along the interface for M93Z2 and M93X2 samples. It can be observed that the M93Z2 sample contained comparatively higher Ni percentage (~average 33%) due to the decreased dissolution in this section of the weld bead. Similar to previous cases, M93X2 showed a smaller Ni percentage than other samples in this section which resulted in an increase in CTE for that sample. Table 3 presents the average Ni weight percentage and resulting CTE of those specific samples. It can be observed that, increasing Ni percentage closer to the optimal value (36%) helps to maintain a lower CTE value in those samples.

In each of the previous sections, element diffusion between the welded alloys was said to be the mechanism responsible for the deviations and increases in sample CTEs. The thermal expansion coefficient of Invar is minimized with a weight percentage of 36% nickel [2]. A slight change in the composition can significantly affect the thermal expansion properties of the alloy. This effect is most noticeable with a decreased nickel percentage where (depending on the temperature that the alloy is studied) a loss of only a couple of percent of nickel can increase the alloy’s CTE far past that of steel [2]. Therefore, since the optimal weight percentage of nickel was altered by diffusion during the welding process, certain sections of the specimen had samples that demonstrated CTEs above those measured from the steel. The effects of the altered material composition were most prevalent near the fusion zone and were not as noticeable on samples taken farther away from the fusion zone. EPMA analysis was performed to provide further explanations on how the composition and the CTE of the samples was affected by diffusion.

### 3.2. Chemical Composition Study Using Electron Probe Micro Analyzer (EPMA)

**Figure 13:**
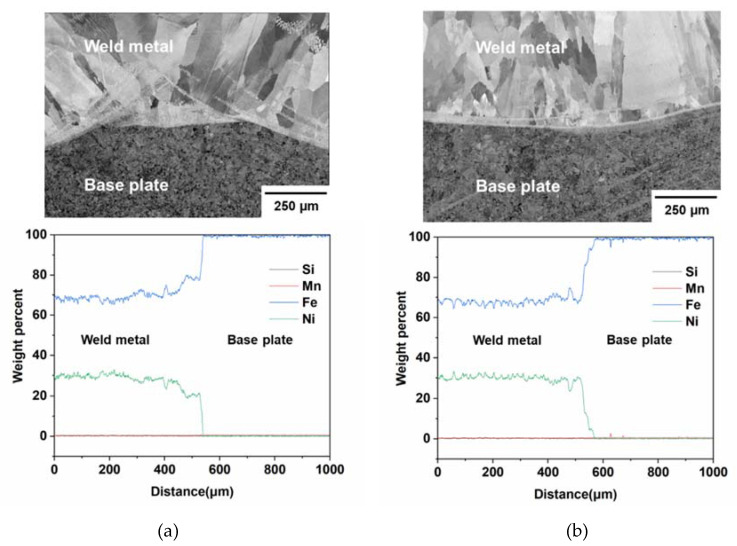
Back-scattered electron image and electron probe micro analyzer (EPMA) line scan signals of (**a**) M93A36Z2 and (**b**) M93Z2 samples.

Although energy-dispersive X-ray spectroscopy could reveal the key mechanism acting behind observed differences in CTE of different samples, EPMA analysis was conducted to validate that trend of elemental dissolution during the AM process. Back-scattered electron (BSE) images along with corresponding EPMA scan signals are presented in Figure 13. Two samples (M93A36Z2 and M93Z2) with same orientation (Z direction) but from two different sections (sections 2 and 3) were selected for this study. Note that these two samples showed a significant difference in their CTE numbers. Figure 13 shows that the M93A36Z2 sample had comparatively lower Ni and higher Fe compared to that of the M93Z2 sample. As a result, the gap between Ni and Fe signals in the plot is wider for the M93A36Z2 sample as compared to that of the M93Z2 sample (refer to EPMA signals in the plots of Figure 13). Higher dissolution of Fe in the M93A36Z2 sample resulted in an increase in CTE and vice-versa for the M93Z2 sample. Also, the Ni percentage started dropping and the Fe percentage started increasing from around 300 µm before the actual interface region for the M93A36Z2 sample. On the other hand, both Ni and Fe percentages were more stable and uniform until very close to the interface region for the M93Z2 sample. This also indicates that the dissolution happened in the M93A36Z2 sample more severely as compared to the M93Z2 sample resulting in a significantly higher CTE for that sample.

### 3.3. Surface Expansion

The CTE of the machined Invar surface is dependent on the expansion of both the Invar and the steel baseplate underneath the Invar beads. Since the CTE of steel is greater than that of Invar, it was expected that the measured CTE would be between the known values for A36 steel and Invar M93. The CTEs measured from the machined Invar surface and the steel baseplate are shown in Table 4. The strain gauge labels indicate the positioning of the gauge and correlate with the locations pictured in Figure 5.

The average CTE on the surface of the machined Invar was 6.57 ± 1.72 μm/m-K. Variations between the measured CTEs are distinguishable between the orientations of the gauges. The two gauges placed along the long dimension of the test specimen measured a larger thermal expansion than the gauges placed along the short direction. The CTEs measured from the steel baseplate maintain consistent magnitudes in both directions. SG1 and SG3 are oriented along the width of the specimen (refer to Figure 5) and have equal lengths of Invar and steel along this orientation. SG2 and SG4 are oriented along the length of the specimen where there is a larger length of steel than Invar. The larger portion of steel along the length of the specimen contributes to the higher CTE measured by gauges oriented in this direction. Overall, the thin layer of machined Invar decreased the surface expansion of the part by about 42%. 

Additional experimentation was performed to measure the CTE of the reference plate and the low-carbon steel plate to further confirm the results of the strain gauge measurements. The measured CTE values for the reference plate and the low-carbon steel are shown in Table 5.

The 1.82 μm/m-K CTE measured for the reference plate is between values 1.2 μm/m-K and 2.7 μm/m-K specified by the manufacturer and ASTM standard F1684-06 [3]. Additionally, the measured CTE for low-carbon steel plate is within the known range for steel of 11 μm/m-K to 12.5 μm/m-K [12].

## 4. Conclusions

This study utilized a fiber-laser welder to print Invar beads on a steel baseplate. The steel, Invar, and the interface between these two materials were analyzed to investigate the thermal expansion characteristics of the printed specimen. An additional experiment was performed to measure the surface expansion of a machined specimen composed of the two materials. The key findings of this study are as follows:

- Steel material under printed Invar maintains thermal expansion properties comparable to those of normal steel. There were minor deviations from the expected CTEs that were seen in samples taken from the ends of weld beads. The expected cause of these dissimilarities is the increased elemental diffusion associated with longer durations of heat exposure.
- The fusion zone between the steel and the Invar had the highest average CTE. The effects of diffusion were most noticeable in the fusion zone region, and the composition of the region was altered to produce undesirable thermal expansion characteristics. Variation in weight percent of nickel due to dissolution showed direct correlation with the resulting CTE of different samples.
- The samples taken from the Invar beads showed a decreased thermal expansion compared to the other sections. The diffusion was noticeable in this region, but it did not increase the sample CTEs past that of steel as seen in the fusion zone region. The effects of diffusion in the Invar region are seen through the decreases in CTE along the length of the region. The sections of this region that are were exposed to heat for a longer time had more diffusion and a larger CTE. Two layers of Invar were required to reduce to reduce the CTE of the specimen.
- The surface expansion of the machined Invar beads was between the expected expansion values for the Invar beads and the substrate. The expansion on the surface of the thin layer of Invar was 42% less than the expansion on the surface of the steel.
- Multi-material tooling with Invar is a viable option to decrease the CTE for tools. Future work should focus on determining the printed thickness of Invar needed to optimize the decrease in thermal expansion.

## Figures and Tables

**Table 1:** Chemical composition of Aperam Invar^®^ M93 wire (wt.%).

Ni	Fe	Mn	C	Si	S	P
36	Balance	0.2–0.4	≤0.04	≤0.25	≤0.0015	≤0.008

**Table 2:** Average and sample coefficient of thermal expansion (CTE) values from thermomechanical analysis.

Section	CTE (μm/m-K)						
	X1	X2	Y1	Y2	Z1	Z2	Average
A36	11.92	12.21	12.96	12.67	11.34	12.53	12.27
WA36	13.06	12.24	12.44	12.56	13.95	11.90	12.69
M93A36	12.14	13.14	12.82	12.08	12.81	15.14	13.02
M93	10.40	9.07	9.03	9.08	6.46	2.09	7.69

**Table 3:** CTE versus weight percentage of nickel.

Sample	Percent Ni	CTE (μm/m-K)
M93X1	30%	10.38
M93Z2	33%	2.11
WA36X1	29%	13.59
M93/A36Z2	28%	15.59

**Table 4:** Strain and CTE of machined Invar beads and steel.

Strain Gauge	Test Material	Change in Strain from 40 °C–160 °C (μm/m)	CTE (μm/m-K)
SG1	Invar	529.22	5.62
SG2	Invar	759.17	7.54
SG3	Invar	417.51	4.68
SG4	Invar	866.02	8.43
SG5	A36 Steel	1227.21	11.44
SG6	A36 Steel	1213.55	11.33

**Table 5:** Strain and CTE of reference specimen and steel plate.

Strain Gauge	Test Material	Change in Strain from 40–160 °C (μm/m)	CTE (μm/m-K)
SG7	Reference Invar	73.82	1.82
SG8	Low-carbon Steel	1283.04	12.00
